# Artificial intelligence for precision management of epithelial ovarian cancer: a comprehensive review

**DOI:** 10.3389/fmed.2025.1713629

**Published:** 2026-01-13

**Authors:** Qing Liu, Chunhua Zhang, Peiquan Li, Ruiyi Jing, Lei Bi, Weiping Chen

**Affiliations:** 1 Faculty of Chinese Medicine, Macao University of Science and Technology, Macao, China; 2 Department of Gynecologic Oncology, Renji Hospital, Shanghai Jiao Tong University School of Medicine, Shanghai, China; 3 Department of Obstetrics and Gynecology, The Second Affiliated Hospital of Nanjing Medical University, Nanjing, Jiangsu, China; 4 School of Chinese Medicine, Nanjing University of Chinese Medicine, Nanjing, Jiangsu, China

**Keywords:** artificial intelligence (AI), epithelial ovarian cancer (EOC), multimodal data integration, prediction of prognosis, treatment

## Abstract

Epithelial ovarian cancer (EOC) has a high rate of incidence and mortality, seriously threatening women’s health. Artificial intelligence (AI) possesses functions such as image recognition, data mining and pattern recognition, which can solve problems that traditional statistical methods cannot handle, such as large amounts of data and data missing. It has achieved breakthrough progress in the fields of risk prediction, diagnosis, treatment and response assessment of malignant tumors. Most AI technologies are mainly applied in the preoperative diagnosis of EOC, as well as in imaging and pathological genomics. However, their application in treatment and prognosis assessment studies is relatively limited. This article reviews the AI application in the treatment and prognosis assessment of EOC in recent years, including the establishment of prediction models for complete cytoreduction (R0 resection), the prediction of chemotherapy and targeted drug efficacy, and the application of different AI technologies based on pathology, radiomics, and clinical data for the prognosis assessment of EOC, with the aim of providing more ideas for the application of AI in EOC.

## 1 Introduction

Ovarian malignant tumors are one of the three major malignant tumors in the female reproductive system, with a mortality rate second only to cervical cancer. According to the statistics in 2020, globally, 314,000 new cases of ovarian cancer were reported each year, about 207,000 deaths occurred each year; in China, the new cases were approximately 62,000 per year, and the deaths were about 33,000 per year (1, 2). Epithelial ovarian cancer (EOC) accounts for more than 80% of ovarian malignant tumors. Due to the lack of effective early screening methods, more than 70% of patients present at the advanced stage (FIGO III-IV stage) when they seek medical treatment, resulting in poor treatment outcomes and a high recurrence rate. The 5-year survival rate is approximately 40%. Standard treatment for ovarian cancer includes standardized surgery, platinum-based combination chemotherapy, and indicated maintenance therapy. However, more than 70% of patients with advanced-stage ovarian cancer experience recurrence after initial treatment. As the platinum-free interval shortens continuously, it eventually develops into platinum-resistant recurrence. Developing personalized treatment plans for patients with EOC can reduce the occurrence of platinum-resistant recurrence, extend the recurrence time, and have positive clinical significance for improving disease prognosis.

The aggressive nature and high recurrence rate of EOC are underpinned by complex and interconnected biological processes. These include extensive angiogenesis, which fosters tumor growth and metastatic spread; metabolic reprogramming that allows cancer cells to adapt and thrive in harsh microenvironments; the enrichment of cancer stem cells that drive therapy resistance and disease relapse; and dynamic interactions within the tumor microenvironment (TME) that promote immunosuppression and tumor progression (3–5). Furthermore, the role of regulatory molecules such as non-coding RNAs adds another layer of complexity to the disease’s pathogenesis and progression. The profound heterogeneity and multifactorial nature of these drivers pose significant challenges for conventional statistical methods, which often struggle to integrate and model such high-dimensional data effectively. This biological complexity, however, creates a compelling rationale for the application of artificial intelligence (AI). AI techniques, particularly machine learning and deep learning, are uniquely suited to decipher intricate, non-linear patterns from large-scale, multi-modal datasets—encompassing genomic, transcriptomic, imaging, and clinical data. Therefore, AI presents a powerful and promising approach to unravel the complexities of EOC, ultimately aiming to improve patient outcomes through enhanced prediction and personalization of therapy.

Artificial intelligence (AI) (6) is a discipline that simulates human intelligence using computers. Machine learning (ML), a core subset of AI, excels at identifying complex patterns within structured data (e.g., clinical variables) to create predictive models. For the multi-faceted data challenges in EOC, deep learning (DL), a more advanced form of ML, is particularly powerful. DL uses multi-layered artificial neural networks to automatically learn hierarchical features directly from unstructured data, such as medical images, without relying heavily on manual feature engineering. These inherent capabilities – processing large amounts of heterogeneous biomedical data, handling missing values, and efficiently analyzing new information – make AI particularly suited for prognostic analysis and treatment planning in EOC. This capability is crucial for analyzing the intricate patterns present in EOC histopathology slides and radiological scans. Consequently, AI is particularly suited for prognostic analysis and treatment planning in EOC due to its ability to process large amounts of heterogeneous biomedical data, handle missing values, and efficiently analyze new information.

This article reviews AI application in EOC’s treatment and prognosis assessment, with the aim of providing more ideas for applicating AI in EOC.

## 2 Review methodology

To ensure a comprehensive and reproducible collection of relevant literature, a systematic search strategy was employed. The primary electronic databases utilized for this review were PubMed, IEEE Xplore, and Web of Science, chosen for their extensive coverage of biomedical, technical, and interdisciplinary research. The literature search was conducted for articles published up to June 2024.

The search strategy was built around a combination of keywords and Medical Subject Headings (MeSH) terms related to the population, intervention, and context. The core search string included: (“epithelial ovarian cancer” OR “EOC” OR “high-grade serous ovarian cancer” OR “HGSOC”) AND (“artificial intelligence” OR “AI” OR “machine learning” OR “deep learning” OR “neural network”) AND (“treatment” OR “prognosis” OR “surgery” OR “cytoreduction” OR “chemotherapy sensitivity” OR “platinum resistance” OR “targeted therapy” OR “survival prediction”). This string was adapted to the specific syntax of each database.

### 2.1 Inclusion and exclusion criteria

Studies were included if they: (1) primarily focused on human subjects with EOC; (2) investigated the application of AI/ML models for purposes related to treatment (e.g., prediction of surgical outcome, chemotherapy or targeted therapy response) or prognosis evaluation (e.g., overall survival, recurrence risk); and (3) were published in English as original research articles.

Conversely, studies were excluded if they: (1) focused solely on diagnostic imaging or pathological classification without a direct link to therapeutic decision-making or prognostic assessment; (2) were review articles, editorials, conference abstracts (unless they provided sufficient methodological and results detail), or non-English publications; (3) did not involve a distinct AI/ML methodology.

### 2.2 Study selection and data extraction

The study selection process involved two stages. First, all retrieved records were screened by title and abstract against the inclusion and exclusion criteria. Second, the full text of potentially relevant articles was thoroughly reviewed to make a final inclusion decision.

From each included study, key information was systematically extracted into a standardized form. This included: (1) first author and publication year; (2) study aim/application; (3) dataset characteristics (sample size and data modality, e.g., CT, histopathology, clinical variables); (4) the specific AI model or algorithm used; and (5) the primary performance metrics reported (e.g., AUC, accuracy, C-index). The findings from this extraction process are synthesized narratively in the following sections.

### 2.3 A conceptual overview of AI workflow

**Figure 1:**
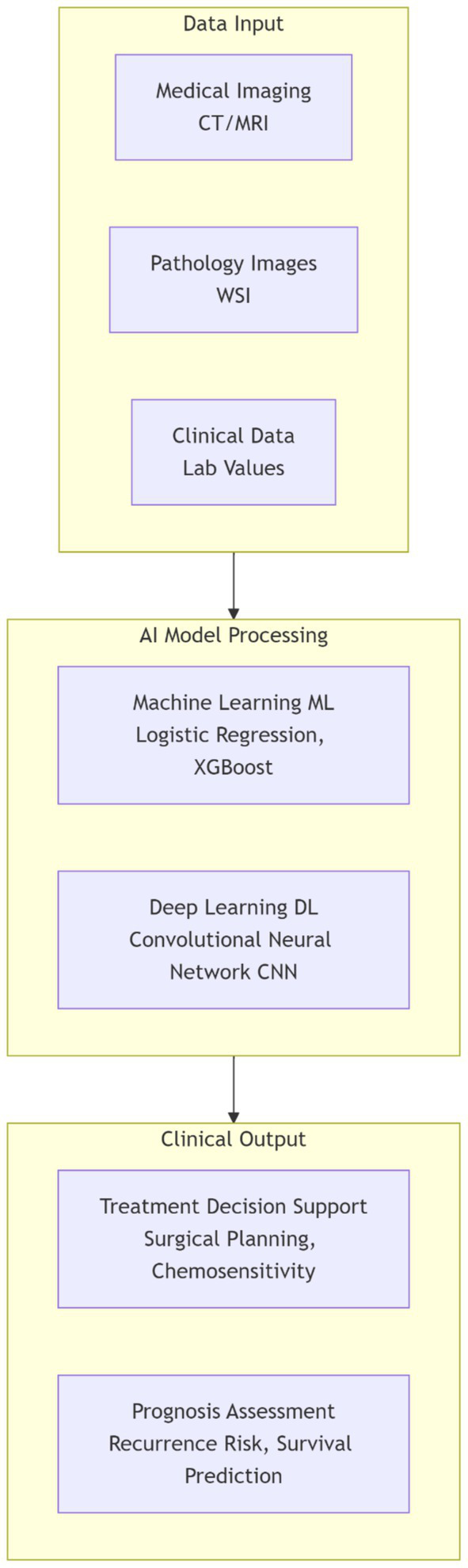
AI workflow for treatment and prognosis in epithelial ovarian cancer (EOC). This schematic illustrates the foundational pipeline for applying artificial intelligence in EOC. Diverse data modalities—including medical imaging, digital pathology, and clinical records—serve as input. These data are processed by specific AI paradigms: machine learning (ML) models are often applied to structured clinical data, while deep learning (DL) models, particularly convolutional neural networks (CNNs), are adept at analyzing image data. The final output provides actionable insights for clinicians, aiding in treatment decision support (e.g., predicting surgical outcomes or drug response) and prognosis assessment (e.g., estimating recurrence risk).

This schematic outlines the foundational pipeline for applying artificial intelligence (AI) in EOC. Diverse data modalities—including medical imaging, digital pathology, and clinical records—serve as the input. These data are processed by specific AI paradigms: Machine Learning (ML) models are often applied to structured clinical data, while Deep Learning (DL) models, particularly Convolutional Neural Networks (CNNs), are adept at analyzing image data. The final output provides actionable insights for clinicians, aiding in treatment decision support (e.g., predicting surgical outcomes or drug response) and prognosis assessment (e.g., estimating recurrence risk) (see Figure 1).

## 3 Application of AI in EOC treatment

The EOC treatment has entered a comprehensive holistic treatment model. Surgery, chemotherapy, and targeted therapy are the main methods for EOC treatment. The treatment outcome is closely related to the satisfaction of the surgery, the size and location of the residual lesions, the formulation of the chemotherapy plan, the BRCA gene status, and the homologous recombination deficiency (HRD) state. Therefore, preoperative thorough assessment of the resection rate of the surgery, early prediction of the sensitivity to chemotherapy and targeted therapy, prediction of the gene mutation status, and screening of platinum-resistant patients are crucial for the formulation of the treatment plan for patients in the comprehensive treatment group.

### 3.1 Prediction of surgical outcome: R0 resection and complete cytoreduction by AI

Surgery is a fundamental treatment measure for ovarian cancer. The initial tumor cell eradication surgery that achieves complete cytoreduction (with no macroscopic residual lesions, R0) is one of the key steps in improving patient prognosis and reducing recurrence. Therefore, preoperative precise assessment of complete cytoreduction can increase the tumor resection rate and improve survival rate. AI can process large amounts of quantitative data from imaging or clinical, and through data processing, build predictive models. DiSilvestro et al. (7) used a computer-aided detection (CAD) algorithm, which often leverages convolutional neural networks (CNNs)—a class of deep learning models specifically designed for processing pixel data. CNNs use layers of filters to automatically extract hierarchical features from images, such as textures and shapes in CT scans, mimicking visual processing. This makes them exceptionally well-suited for analyzing medical images to establish a set of CT features that can prognosticate complete cytoreduction, improving the accuracy of predicting surgical outcomes. In addition to imaging assessment, some studies have also included clinical indicators to establish predictive models for complete cytoreductive surgery using artificial intelligence technology. Laios et al. (8) applied the k-nearest neighbor (k-NN) classifier to anticipate the performance of R0 surgery in advanced ovarian epithelial cancer and compared it with logistic regression. The researchers selected patients diagnosed with advanced, high-grade serous ovarian cancer, fallopian tube cancer and primary peritoneal cancer from the ovarian database and who underwent surgical cytoreductive surgery from 2015 to 2019. The variables included age, body mass index (BMI), disease score, Charlson Comorbidity Index (CCI), operation time and surgical complexity. In this study, the k-NN model’s performance was primarily evaluated on the training set, with a reported average accuracy of 66%. While the authors note that this approach demonstrated superior performance compared to logistic regression within their dataset, it is crucial to contextualize this result: an accuracy of 66% is quite low and falls substantially below the threshold considered viable for reliable clinical decision-making. This finding underscores that while k-NN may offer a statistical improvement over simpler benchmarks in certain contexts, its predictive power in this application is not yet sufficient for clinical adoption. Consequently, this work highlights a clear need for the development and validation of more robust and accurate predictive models for this task. In 2022, (9) a retrospective cohort study was conducted on 571 patients with advanced EOC who underwent cytoreductive surgery. Based on clinical data such as patient age, stage, intraoperative tumor distribution, and peritoneal involvement, a model for predicting complete cytoreduction for ovarian cancer was developed. This model employed the computer extreme gradient boosting (XGBoost) algorithm, and SHAP (Shapley Additive Explanations) was used for the overall and local interpretation of the model. The model could demonstrate robust performance in internal validation for predicting the R0 resection rate [area under the curve (AUC) = 0.866; 95% confidence interval (CI) = 0.8–0.93]. This study not only demonstrated robust performance of the model in predicting R0 resection for EOC patients, but also identified the “threshold points” for increasing the probability of satisfactory cell ablation surgery, including intraoperative ovarian cancer map score <5, surgical complexity score >4, peritoneal tumor index <5, patient age <60 years old, maximum tumor volume <5 cm. Additionally, Laios et al. (10) also utilized interpretable artificial intelligence to explore the potential role of upper abdominal peritoneal resection in achieving complete cytoreduction in advanced ovarian cancer. This study utilized the structured ESGO ovarian cancer reporting template and employed the XGBoost algorithm to simulate a long series of surgical sub-procedures and utilized the SHAP framework to provide global (queue) interpretable results. The results showed that the model predicted complete cytoreduction with acceptable accuracy (AUC = 0.70; 95% CI = 0.63–0.76), and it was concluded that upper abdominal peritoneal resection (UAP) is the most important feature for predicting complete cytoreduction surgery in ovarian cancer. Complementing these studies, a very recent multi-center study developed a hybrid DL model that integrates both pre-operative CT scans and standard clinical laboratory values to predict the likelihood of complete cytoreduction (11). Their model, validated on a large cohort of over 800 patients from three independent institutions, achieved an AUC of 0.89, demonstrating superior generalizability and highlighting the power of combining deep image features with readily available clinical data.

#### 3.1.1 Synthesis and key challenges

The studies reviewed herein demonstrate a collective effort to preoperatively predict complete cytoreduction (R0) in EOC. A common theme is the integration of preoperative CT imaging features with key clinical and surgical variables such as the peritoneal cancer index, surgical complexity score, and patient age. In terms of algorithmic choice, while traditional methods like k-NN have been explored, more advanced ensemble methods (e.g., XGBoost) coupled with interpretable AI frameworks (e.g., SHAP) are emerging as powerful tools. These models not only achieve promising predictive performance, with AUCs ranging from approximately 0.70 to 0.87, but also provide crucial clinical insights by identifying the most influential predictive features (e.g., upper abdominal procedures, tumor distribution scores).

However, several challenges persist. The reported performance varies, and some models, while outperforming logistic regression, still exhibit accuracy (e.g., 66%) that is insufficient for standalone clinical application. This variability can be attributed to differences in study populations, surgical standards, and the specific set of features used. A significant limitation across the field is the lack of robust external validation on independent, multi-centric cohorts. Furthermore, the clinical integration of these models requires standardization of both the input variables (e.g., consistent surgical scoring systems) and the AI platforms themselves.

### 3.2 Prediction of chemotherapy sensitivity by AI

Platinum-based chemotherapy is the cornerstone of ovarian cancer treatment. The responsiveness of patients to platinum directly determines the efficacy of chemotherapy. However, there are currently no effective biomarkers for predicting the response to platinum-based treatment. BRCA1/2 mutations and HRD status are currently the most commonly used biomarkers for evaluating the therapeutic effects of platinum-based chemotherapy and PARP inhibitors. However, the detection is costly, time-consuming, and has poor accessibility. Bourgade et al. (12) developed a deep learning (DL) model that predicts the BRCA mutation status of patients with high-grade serous ovarian carcinoma (HGSOC) through whole-slide images (WSIs) of hematoxylin–eosin (HE) staining. This approach is powerful because DL can discern subtle, morphologic patterns in the vast amount of pixel data within WSIs that are imperceptible to the human eye, effectively linking histological phenotypes to genomic alterations. The AUC of this model for predicting BRCA mutation status in the test set and validation set was 0.681 and 0.631, respectively. Moreover, the relevant information of BRCA mutations is mainly located within the tumor background and has a recognizable phenotypic effect, suggesting that this learning model can be used as a pre-screening tool. Bergstrom et al. (13) developed a deep learning platform named DeepHRD for predicting HRD from pathological slides. Through transfer learning from HGSOC, DeepHRD predicted that HRD patients had better overall survival rates after first-line treatment and platinum-based neoadjuvant therapy. Compared with molecular testing, the overall survival of HRD patients classified by DeepHRD was 1.8 to 3.1 times longer. Besides predicting genes or HRD status, it is also possible to predict the sensitivity to chemotherapy by combining radiomics or pathomics with clinical features. Li et al. (14) selected radiomics features from MRI of advanced HGSOC patients using a recursive feature elimination method grounded on support vector machines. They established a nomogram to foretell platinum-resistant models for advanced HGSOC using radiomics features and three clinical features (FIGO stage, CA-125, residual tumor). Results showed that this model’s AUC was 0.799, higher than 0.747 of the single clinical model. The model predicted a shorter progression-free survival for high-risk groups compared to low-risk groups, suggesting that the MRI-based radiomics nomogram has the potential to recognize platinum resistance and is helpful for individualized management of advanced HGSOC. The model developed by Li et al. demonstrated superior performance for predicting platinum resistance compared to a model based on clinical features alone, achieving a higher area under the curve (AUC of 0.799 versus 0.747). Furthermore, the radiomics nomogram showed significant prognostic value by successfully stratifying patients into distinct progression-free survival groups in their cohort, and the radiomic score was also associated with chemotherapy response and surgical outcome. Ahn et al. (15) developed the PathoRiCH model for HGSOC pathological risk classification based on histopathological images. This model predicted that patients of platinum-responsive group and platinum-unresponsive group showed prominently different platinum-free intervals in an internal cohort and two independent external cohorts, demonstrating better predictive performance than present molecular biomarkers; associating PathoRiCH with molecular biomarkers showed even better predictive performance. Moving beyond single data modalities, Kilim et al. (16) proposed a novel multi-modal fusion framework. Their model concurrently processes H&E-stained whole-slide images and genomic data to predict platinum sensitivity, outperforming models based on either modality alone (AUC: 0.82 vs. 0.75 and 0.78). This work underscores the emerging trend and significant advantage of integrating disparate data types for more accurate predictions. Nakayama et al. (17) research indicated that the use of AI-assisted ML clustering for classifying EOC patients can determine the platinum sensitivity rate and disease recurrence rate, which is helpful for physicians in formulating treatment plans and evaluating prognosis.

#### 3.2.1 Synthesis and key challenges

The application of AI for predicting chemotherapy sensitivity in EOC is rapidly evolving, primarily focusing on inferring platinum response. The research showcases a paradigm shift from relying solely on costly and time-consuming molecular tests towards using more readily available data. Two prominent approaches are: (1) using AI to predict the status of established biomarkers (like BRCA mutations and HRD) directly from H&E-stained pathology slides and (2) building direct predictive models using radiomic features from MRI or pathomic features from histology images, often combined with clinical data.

The performance of slide-based models for predicting BRCA/HRD status is modest but promising for pre-screening (AUCs ~0.63–0.68), while models combining radiomics/pathomics with clinical features show higher accuracy (AUCs ~0.79–0.80) for predicting platinum resistance directly. A key finding is that pathomic and radiomic features capture biological information beyond known biomarkers, as evidenced by their ability to stratify patient survival independently. The integration of AI-derived scores with molecular biomarkers (e.g., PathoRiCH) appears to be the most promising direction, yielding superior predictive power.

Critical challenges include the interpretability of these “black-box” models—understanding why a prediction is made is crucial for clinical adoption. Additionally, the generalizability of pathomic models needs to be firmly established across different pathology preparation protocols and scanners. The ultimate goal is the development of robust, multi-modal models that can provide a comprehensive assessment of tumor behavior and treatment sensitivity.

### 3.3 Prediction of the effect of targeted drugs by AI

Bevacizumab is a molecular targeted drug for treating advanced ovarian cancer. However, this drug is expensive and has potential adverse reactions such as hypertension, proteinuria, gastrointestinal perforation, and thromboembolic events. Not all patients respond well to the treatment. Therefore, predicting the patients’ treatment responsiveness is of crucial importance for formulating precise treatment plans. Wang et al. (18) developed a weakly supervised deep learning model and hybrid deep learning framework using four immunohistochemical tissue samples (AIM2, c3, C5, and NLRP3) to predict bevacizumab’s treatment responsiveness in ovarian cancer patients. This model, combined with AIM2, achieved a prediction accuracy of 0.92 in the first experiment (34% testing and 66% training), 0.86 in the second experiment (five-fold cross-validation). Cox proportional hazards model and Kaplan–Meier PFS analysis further identified that the AIM2-DL model can distinguish patients who can achieve positive treatment outcomes after treatment.

### 3.4 Application of AI in new drug development

Beyond guiding established therapies, AI holds promise for accelerating drug discovery and development in EOC. While direct examples of AI-discovered EOC drugs in the clinic are still emerging, the approach is being actively explored. AI can analyze multi-omics data (e.g., genomics, transcriptomics) from EOC tumors to identify novel therapeutic targets and biomarkers. For instance, AI models can predict the efficacy and synergism of existing or investigational drug combinations by leveraging large-scale drug screening data integrated with genomic features of EOC cell lines (19). Furthermore, in the realm of drug repurposing, AI-driven analysis of molecular signatures and real-world patient data has identified non-oncological drugs with potential anti-tumor activity in EOC, offering a faster route to clinical translation (20). While still in its relative infancy for EOC-specific applications, the integration of AI in these stages of drug discovery presents a paradigm shift towards a more data-driven and efficient approach to developing new therapeutic strategies for ovarian cancer.

## 4 Application of AI in prognosis evaluation of ovarian cancer

Ovarian cancer patients suffer from significant molecular and histological heterogeneity, resulting in the lack of effective prognostic assessment tools. AI can provide a new perspective for prognosis evaluation through the analysis of clinical data, medical images, and pathological images.

### 4.1 Pathological features

Pathological features are one of the more commonly used indicators in the prognosis assessment of ovarian cancer by AI. Binas et al. (21) established a database containing 11,095 MRI images. They calibrated and evaluated the cancerous regions using image processing technology and extracted quantitative features using two specific MRI imaging sequences. By applying AI to define heterogeneous regions of tumor cells based on an apparent diffusion coefficient (ADC) cutoff value of 3 mm^2^/s, their automatic classification system achieved an average classification accuracy of 0.86. This result suggests that the AI model can be a valuable tool for assessing tumor heterogeneity from standard clinical MRI scans. Yang et al. (22) constructed a deep learning model for predicting the response to adjuvant therapy and prognosis of ovarian cancer patients using HE-stained WSIs from the TCGA-OV cohort. They developed a deep learning model incorporating prognostic background information from pathological images – the Ovarian Cancer Digital Pathological Index (OCDPI). Multivariate analysis indicated that OCDPI was an independent prognostic factor. Patients with a low OCDPI had a better prognosis, longer progression-free and overall survival, and a lower recurrence rate after adjuvant therapy.

### 4.2 Imaging features

Imaging is also frequently used to assess the prognosis of ovarian cancer. Wei et al. (23) developed a nomogram based on the radiomics features of preoperative enhanced CT in HGSOC, combined with clinical variables to prognosticate the risk of recurrence. Results disclosed that the radiomics features had an accuracy of 82.4, 77.3, and 79.7% for predicting the 18-month recurrence risk in the training cohort, internal validation cohort, independent external validation cohort, respectively. The discrimination accuracy for 3-year recurrence risk was 83.4, 82.0, and 70.0%, respectively. The radiological nomogram’s accuracy in foretelling 18-month and 3-year recurrence risks was 84.1 and 88.9%, respectively. In the realm of radiomics, a recent study by Yao et al. (24) leveraged pre-treatment CT images to predict not just recurrence risk, but also the likely anatomical site of recurrence (e.g., peritoneal, distant). Their CNN-based model achieved an 81% accuracy for site-specific recurrence prediction, providing clinically actionable insights for personalized surveillance strategies.

### 4.3 Clinical data

Clinical data, due to their abundant resources and relatively easy accessibility, have been increasingly applied by AI to prognostic assessment models in recent years. Feng et al. (25) established a machine learning model using clinical and hematological indicators of EOC patients, employing the decision tree method in ML to anticipate the survival of EOC patients. The results showed that lymphocyte ratio (LY%), monocytes/lymphocytes (MO/LY), CA125 level, differentiation status, ascites cytology, NE, and age could be used to predict survival, and MO/LY was the root node of the decision tree, with a survival prediction AUC of 0.69. Additionally, the survival prediction AUC of logistic regression (LR) was 0.55 (95% CI, 0.53–0.57), indicating that the decision tree’s performance was superior to that of logistic regression. In 2021, Ma et al. (26) randomly assigned serum tumor markers (including TCT) to training and validation groups, and used eight machine learning classifiers to establish models to evaluate efficacy. The results showed that machine learning techniques, particularly the random forest classifier, outperformed traditional regression analysis in foretelling several clinical parameters associated to EOC. Liu et al. (27) developed a diagnostic and prognostic prediction model, DeepCox, based on the characteristic that thrombocytosis in ovarian cancer patients is associated with shortened overall survival, using tumor-tuned platelet RNA. This model can demonstrated robust performance in identifying patients with higher recurrence risk and shorter survival periods, and this model is independent of risk factors such as disease stage and residual lesion size that affect patient prognosis. For recurrent ovarian cancer, complete secondary cytoreduction may improve prognosis. Bogani et al. (28) analyzed using artificial neural networks (ANN) and found that the three main factors that promote satisfactory tumor cell debulking were disease-free interval, retroperitoneal recurrence, and residual disease at the time of the initial surgical treatment. Among these, the disease-free interval was the most important factor for predicting satisfactory tumor cell debulking and overall survival.

### 4.4 Synthesis across data modalities

As reviewed in the preceding sections, AI models for EOC leverage diverse data types, each offering unique advantages and facing specific limitations. Pathomics provides high-resolution insight into the tumor microenvironment and cellular architecture, making it a powerful tool for prognostic stratification and predicting therapy response. Radiomics offers a non-invasive, macro-scale view of entire tumors and their heterogeneity, which is invaluable for assessing disease spread and recurrence risk. Clinical data, being universally available, facilitates the development of accessible models that capture the patient’s systemic status and are readily integrable into clinical workflows.

A critical appraisal, however, reveals that no single modality is sufficient to capture the full complexity of EOC. The choice of data source entails inherent trade-offs between biological resolution, clinical invasiveness, cost, and scalability. Table 1 provides a comparative overview of these data modalities, summarizing their primary applications, representative AI models, key advantages, and major limitations.

**Table 1:** Comparison of data modalities in artificial intelligence for epithelial ovarian cancer.

Data modality	Primary applications	Representative AI models/studies	Key advantages	Major limitations
Radiomics (CT, MRI)	• Predicting surgical resectability (R0) • Forecasting recurrence risk • Predicting platinum sensitivity	• Wei et al. (23) (CT-based nomogram) • Li et al. (14) (MRI-based nomogram)	• Non-invasive • Captures whole-tumor heterogeneity • Readily available in clinical practice	• Lacks cellular/molecular detail • Dependent on imaging protocol and scanner • Features may not be directly biologically interpretable
Pathomics (histopathology WSIs)	• Predicting BRCA/HRD status • Prognostic stratification (e.g., OCDPI) • Predicting chemotherapy response	• Bourgade et al. (12) • Yang et al. (22) (OCDPI) • Ahn et al. (15) (PathoRiCH)	• High-resolution cellular detail • Reveals tumor microstructure and TME • Gold standard for diagnosis	• Invasive (requires biopsy) • Subject to sampling bias • Complex computational requirements • Slide preparation variability
Clinical and hematological data	• Overall survival prediction • Patient risk stratification • Informing treatment decisions	• Feng et al. (25) (decision tree) • Ma et al. (26) (random forest)	• Easily accessible and low-cost • Reflects systemic host response • High clinical relevance and integrability	• May lack direct mechanistic insight into tumor biology • Can be prone to missing data • Limited by the quality of routine data collection
Multi-modal integration (combining two or more modalities)	• Aims to achieve superior, holistic prediction accuracy by capturing complementary biological information	-- (Emerging area, see Prospects section)	• Mitigates limitations of individual modalities • Potential for more robust and generalizable models • Provides a more comprehensive “digital phenotype” of the disease	• Highest data infrastructure complexity • Algorithmic challenges in data fusion and interpretation • Requires large, well-curated, multi-source datasets

TME, tumor microenvironment; WSI: whole-slide image; OCDPI: Ovarian Cancer Digital Pathological Index.

The most promising avenue for future research lies in the multi-modal integration of these complementary data streams (29). By combining the depth of pathomics, the breadth of radiomics, and the context of clinical data, AI models can potentially overcome the limitations of any single approach. Such integrated systems aim to construct a more holistic and accurate digital representation of the tumor and its host, which is essential for advancing personalized medicine in EOC.

## 5 Shortages and prospects

Although AI has demonstrated outstanding performance in aspects such as ovarian cancer imaging diagnosis, pathological diagnosis, treatment, and prognosis prediction, its translation into clinical practice faces several significant hurdles. A paramount challenge, beyond the mere need for large sample sizes, is the monumental effort required for data curation and annotation. The development of highly accurate models, particularly for image analysis, often depends on pixel-level annotations on pathology slides or precise segmentation of tumors on CT scans, which must be performed by specialized pathologists and radiologists. This process is expensive, time-consuming, and a known source of label noise due to inter-observer variability, creating a critical bottleneck that limits the pace and scale of AI research in EOC.

To overcome this data curation bottleneck, there is a growing focus on developing AI methods that can learn from less extensively annotated data. Weakly-supervised learning represents a particularly promising direction. These algorithms can effectively train models using only slide-level or patient-level labels (e.g., “BRCA mutant” or “platinum-resistant”) instead of requiring exhaustive, pixel-level manual outlines. For instance, the study by Wang et al. (18) mentioned in this review exemplifies this approach, where a weakly-supervised deep learning model was successfully developed to predict bevacizumab response using immunohistochemical samples without the need for detailed, cell-by-cell annotations. The advancement of such methods is crucial for leveraging the vast archives of existing, partially labeled medical data and for making the development of new AI models more feasible and efficient.

Beyond data challenges, it is difficult for people to fully understand the underlying biological processes hidden behind AI models, reducing the interpretability of AI algorithms. AI also faces challenges such as data privacy, algorithm transparency, and ethical issues, which require further improvement in medical ethics, regulatory requirements, etc.

However, it is expected that with the integration of large-scale multi-center, prospective data collection and multimodal data, the application of reinforcement learning and transfer learning, optimization of deep learning models, and the use of interpretable AI technologies, AI can eventually be truly applied in the clinical environment and better serve clinical practitioners and patients.

**Figure 2:**
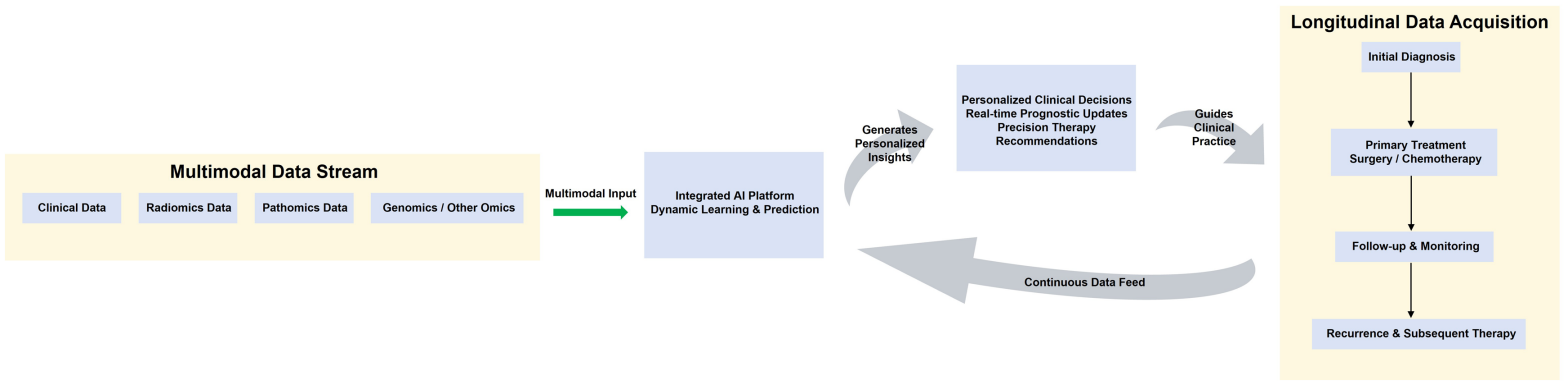
Future vision: integrated, multimodal AI and the MEDomics paradigm in EOC. The future of EOC management lies in the MEDomics paradigm, which involves the longitudinal acquisition of multimodal data throughout the patient’s disease course (top timeline). Clinical, radiomic, pathomic, and genomic data streams are continuously fed into an Integrated AI Platform. This platform employs dynamic learning models that update their predictions as new data becomes available. The output consists of real-time, personalized clinical decisions, which in turn guide the next steps in patient management, creating a closed-loop, adaptive system for precision oncology.

Looking further ahead, the field is moving towards the paradigm of MEDomics (Multimodal Electronic Data and OMICS), which emphasizes the longitudinal collection of comprehensive clinical, imaging, pathological, and multi-omics data over a patient’s entire disease journey (30, 31). For a dynamic and evolving disease like EOC, where treatment responses shift and recurrence risks change over time, this longitudinal perspective is critical. The future of AI in EOC lies not only in sophisticated one-time models but also in developing adaptive AI systems that can continuously learn from this streaming MEDomics data. Such systems would be capable of updating prognostic and predictive assessments in real-time, moving beyond static snapshots to provide a dynamic, lifelong learning companion for clinical decision-making. This AI-driven, integrative framework promises to unlock a new era of personalized and pre-emptive management for patients with EOC, ultimately guiding therapy selection at every stage of the disease with unprecedented precision. This envisioned future, integrating the MEDomics paradigm with dynamic AI, is illustrated in Figure 2.
